# Management and prognostic analysis of patients with gestational trophoblastic neoplasia (GTN) in FIGO stage IV and its special type

**DOI:** 10.1007/s10585-020-10064-w

**Published:** 2020-11-20

**Authors:** Kai Wang, Yaxia Chen

**Affiliations:** Department of Oncology, Zhejiang University School of Medicine Women’s Hospital, No. 1 Xueshi Road, Hangzhou, China

**Keywords:** Stage IV GTN, Metastatic lesions, Special type, Prognostic factors, Chemoresponse, Immunohistochemistry (IHC)

## Abstract

GTN is a group malignant diseases from placental trophoblastic cells. There are very few cases of GTN with FIGO (International Federation of Gynecology and Obstetrics) stage IV all over the world, and the special types (patients with metastatic lesions and with no evidence of GTN neither in genitalia nor in lungs) have rarely been reported. It is necessary to conduct large retrospective studies aimed at exploring the diagnosis, treatment and outcomes of this disease. In this retrospective study, 716 patients with GTN were treated at Zhejiang University School of Medicine Women’s Hospital between January 1999 and September 2019; 26 patients were diagnosed as stage IV GTN; Among the 26 stage IV GTN patients, 5 were defined as the special types. The 5-year OS rate of the total 26 FIGO stage IV GTN patients was 69.0%. There was no significant difference of survival rate between stage IV GTN and its special type. And no significant differences in blood type, antecedent pregnancy type, the interval from last known pregnancy, pretreatment serum HCG (human chorionic gonadotropin) level, maximum diameter of tumors, FIGO score, underwent surgery or not and pathological pattern by the outcomes. Age, number of tumor lesions, primary chemotherapy regimen was EMA-CO or EP-EMA protocol and chemoresponse affected the prognosis significantly. Only number of tumor lesions > 8 was independent prognostic factors associated with poorer OS.

**Electronic supplementary material:**

The online version of this article (10.1007/s10585-020-10064-w) contains supplementary material, which is available to authorized users.

## Introduction

GTN is a group malignant diseases from placental trophoblastic cells, including invasive hydatidiform mole, choriocarcinoma, placental site trophoblastic tumor (PSTT) and epithelioid trophoblastic tumor (ETT), the latter two types both are intermediate trophoblastic tumors (ITT). GTN is not common gynecological malignancies, arising from any type of pregnancy, mostly after hydatidiform mole pregnancy, the rest can occur after preterm birth, abortion, term pregnancy and ectopic pregnancy. As the most malignant form, choriocarcinoma usually and early has tumor metastasis, witch is mainly after nonmolar pregnancy. The metastatic sites are variety and individual, lungs are the most common, about 80% patients would happen, followed by vaginal in 30%, pelvic in 20%, liver in 10%, and brain in 10%. According to FIGO staging and classification system for GTN in 2000, tumors extending to the other organs with or without lungs and reproductive organs involvement are classified as stage IV [1, 2]. A Chinese study evaluated this scoring system and concluded that it was effective and closely associated with GTN patients’ prognosis. Meanwhile, they showed that the rate of mortality, recurrence and progression was significantly increasing with the advancement of staging. Patients with metastatic lesions of liver, brain, gastrointestinal tract, spleen, renal and lungs prominently have worse outcomes. There is no significant influence on the prognosis when vaginal or pelvic peritoneum metastases occur [3].

Grossly, patients of GTN present with vaginal bleeding, hysterauxesis, ovarian luteinizing cyst and the symptoms associated with metastases in variable organs, such as local hemorrhage or tumor-mass effects. Sometimes, certain patients see their doctors because of the symptoms related to special metastatic lesions, and there is no evidence of GTN in genitalia or lungs (whether imaging or histopathological examination), such cases are discussed as the “special types” in this study. Theoretically, they may appear when primary focus disappeared and the distant metastatic lesions progressed.

There are very few cases of GTN staging IV all over the world [4–7], and the special types have rarely been reported [8, 9]. Stage IV GTN patients have poorer prognosis caused by single or multiple distant metastases, where death may be linked to age, interval from antecedent pregnancy, antecedent pregnancy type, pretreatment serum HCG level, metastatic sites, number of lesions, FIGO score, surgery or not, pathological pattern, response to chemotherapy and so on. However, some research showed that many risk factors, like older age, antecedent pregnancy, elevated serum β-HCG levels were not significantly associated with survival, in common, they all revealed that multi-agent chemotherapy failure history is the significantly independent risk factor [3, 10–12].

## Materials and methods

### Patients and data collection

This is a retrospective cohort study, aimed at analysing the management, prognosis and the relative risk factors of patients with GTN in FIGO stage IV and its special type. In this study, 716 patients with GTN were treated at Zhejiang University School of Medicine Women’s Hospital between January 1999 and September 2019; 26 patients were diagnosed as stage IV GTN (3.6%); Among the 26 patients, 5 (19.2%) were defined as the special types (patients with metastatic lesions and with no evidence of GTN neither in genitalia nor in lungs). The clinical records were gathered from the institutional database. All of the clinical data were anonymized. The approval for this study was provided by the ethical committee of Zhejiang University School of Medicine Women’s Hospital, Hangzhou, China. Owing to the retrospective study design and analysis of clinical data, written informed consent was formally waived.

### Pretreatment evaluation

The primary diagnosis of stage IV GTN was made according the FIGO 2000 staging system. Pretreatment evaluation consisted of a complete medical history, clinical examinations, serum HCG measurements, routine blood tests, serum biochemical examination, imaging tests. Patients were scored according to the FIGO prognostic scoring system 2000.

### Assessment of treatment and effect

During the treatment, serum HCG level, routine blood test, and serum biochemical examination were monitored regularly for evaluating response and toxicity of chemotherapy. Chest radiograph, Doppler ultrasound scan of abdomen and pelvis or other necessary image examinations were followed regularly. The reference value of serum HCG level for normal results was below 5.3 IU/L. Cytologic and histologic techniques were used to evaluate the lesions from GTN patients treated with surgery, IHC methods were performed on partial specimen sections. By the end of follow-up, there were four treatment outcomes discussed in this research, these statuses were assessed as no evidence of disease (NED), dead, lost to follow up and under treatment. Herein, NED was defined as a condition that there is no evidence of GTN after complete remission (CR), CR refers to consecutive normalization of serum HCG levels for at least 4 weeks after chemotherapy and then at monthly intervals for 1 year. The chemotherapeutic effect was evaluated from three aspects: sensitive, resistant and dependent. Tumor resistance is described as that there is an increase of HCG level > 10% or plateau of ± 10% over a 2 to 3-week period or a decrease of HCG concentrations of less than 15% after a course of chemotherapy and (or) there is development of new metastases [13]. Sensitive response to chemotherapy is empirically described as a decrease of serum HCG concentrations of at least 50% after a cycle of chemotherapy, as Izildinha Maestá et al. [14] had mentioned, HCG level declined by 1 log value over a chemotherapeutic period in chemosensitive patients. Drug dependence is defined as that serum HCG levels drop significantly even down to normal during the chemotherapeutic period, but elevate immediately after drugs suspension. However, no consensus on a defining guideline regarding the diagnosis of GTN chemoresponse has been established.

### Statistical analysis

Statistical analyses were performed using SPSS 20.0 statistical software tool for Windows (IBM Corporation, Armonk, NY, USA). All the variables were classified into qualitative and quantitative indicators before analysis. The Kolmogorov–Smirnov Z test was used to test whether or not the continuous variables is normally distributed. Continuous data were described as mean ± SD (standard deviation) or median (interquartile range/IQR), and categorical data were demonstrated as frequency and percentage. In the univariate analysis, qualitative variables were examined using Fisher’s exact test, and quantitative variables were examined using Student’s *t* test for normally distributed data or using Wilcoxon-Mann–Whitney U test for the non-normal distribution data. Logistic regression method was applied for prediction of independent risk factors. Survival curves were plotted using the Kaplan–Meier method and compared by the log-rank test. Analyses of multivariates were done by Cox proportional hazards regression models. A value of P < 0.05 was considered statistically significant.

## Results

### Patients’ clinical characteristics

In this retrospective cohort of 716 patients, the incidence of patients with FIGO stage IV GTN and its special types was 3.6% (n = 26) and 0.7% (n = 5), respectively. Tables 1 and 2 showed clinical characteristics of the 26 patients in detail. Mainly described treatment outcomes in this study were NED and dead, in 15 patients (57.7%), 7 patients (26.9%), respectively. Besides, there were 2 patients (7.7%) still under treatment and 2 patients (7.7%) lost to follow up. The mean age of patients included was 34.27 ± 9.78 years, and mean NED patients age was 30.53 ± 7.927 years, mean dead patients age was 40.14 ± 9.299 years. The antecedent pregnancy was hydatidiform mole, term delivery, abortion or ectopic pregnancy, in 6 patients (23.1%), 9 patients (34.6%), 9 patients (34.6%) and 2 patients (7.7%), respectively. For 26 stage IV GTN patients, median (IQR) of the interval from last known pregnancy was 9.5 (34) months, 10 (30) months for NED patients and 7 (70) months for dead patients. Pretreatment serum HCG levels were analysed after logarithmic transformation (logHCG). The mean log (HCG concentration) of all the 26 patients, 15 NED patients and 7 dead patients respectively was 4.4588 ± 0.96928 IU/L, 4.3466 ± 1.20156 IU/L, 4.6015 ± 0.53236 IU/L. Given that the FIGO prognostic scoring system was inapplicable to patients with ITT [2]. The mean FIGO score of 12 patients with choriocarcinoma or invasive mole, NED patients and dead patients respectively was 12.50 ± 4.964, 10.00 ± 4.472, 16.75 ± 3.403.

**Table 1:** Clinical features of the 26 GTN patients with distant metastasis treated at Zhejiang University School of Medicine Women’s Hospital from 1999 to 2019

Characteristics	Patients
Age (years)	
< 40	17 (65.4%)
≥ 40	9 (34.6%)
Blood type	
A/+	10 (38.5%)
B/+	6 (23.1%)
AB/+	2 (7.7%)
O/+	6 (23.1%)
Unknown	2 (7.7%)
Antecedent pregnancy	
Molar pregnancy	6 (23.1%)
Term pregnancy	9 (34.6%)
Abortion	9 (34.6%)
Ectopic pregnancy	2 (7.7%)
Interval (months)^a^	
≤ 6	9 (34.6%)
7–12	4 (15.4%)
> 12	13 (50.0%)
HCG level before treatment (IU/L)	
≤ 10^4^	6 (23.1%)
> 10^4^–10^5^	13 (50.0%)
> 10^5^	7 (26.9%)
The maximum diameter (cm) (different imaging tests)	
< 5	14 (53.8%)
≥ 5	12 (46.2%)
Number of tumor lesions	
≤ 8	14 (53.8%)
> 8	12 (46.2%)
Metastatic sites	
Lung	18 (69.2%)
Brain	14 (53.8%)
Hepatic	8 (30.8%)
Gastrointestinal	4 (15.4%)
Spleen	3 (11.5%)
Renal	3 (11.5%)
Pancreas	2 (7.7%)
Adrenal gland	1 (3.8%)
Other distant sites	Gallbladder, pleura, peritoneum, abdominal wall, lymphonodus, skin, muscle, etc.
Lung or reproductive organs with more other organs	21 (80.8%)
Only special sites	5 (19.2%)
FIGO score^b^	
0–6	1 (3.8%)
7–12	5 (19.2%)
≥ 13	6 (23.1%)
Chemoresponse	
Sensitive	15 (57.7%)
Resistant	6 (23.1%)
Dependent	5 (19.2%)
Primary chemotherapy regimen is EMA-CO or EP-EMA	
Yes	18 (69.2%)
No	8 (30.8%)
Surgery	
Yes	19 (73.1%)
No	7 (26.9%)
Pathological pattern	
Choriocarcinoma	9 (34.6%)
ETT	4 (15.4%)
PSTT	2 (7.7%)
Invasive mole	3 (11.5%)
Coexisting	2 (7.7%)
Unknown	6 (23.1%)
Status	
No evidence of disease (NED)	15 (57.7%)
Dead	7 (26.9%)
Under treatment	2 (7.7%)
Lost to follow up	2 (7.7%)

*EMA*-*CO* etoposide, methotrexate, dactinomycin-cyclophospha-mide, vincristine, *EP*-*EMA* etoposide, methotrexate, dactinomycin-etoposide, cisplatin, *IU/L* international units per liter; cm: centimeter

^a^Interval from the termination of last known pregnancy to the diagnosis of GTN

^b^The FIGO prognostic scoring system was inapplicable to patients with ITT

**Table 2:** Univariate correlation analyses of outcomes in gestational trophoblastic neoplasia patients with FIGO stage IV and its special type

Variables/categories	NED (n = 15)			Dead (n = 7)			Mean ± SD/median (IQR)	P value
	N	%	Mean ± SD/median (IQR)	N	%	Mean ± SD/median (IQR)		
Age (years)			30.53 ± 7.927			40.14 ± 9.299	34.27 ± 9.784	0.107
< 40	12	80.00		3	42.86			
≥ 40	3	20.00		4	57.14			
Blood type								0.725
A/+	5	33.33		3	42.86			
B/+	3	20.00		3	42.86			
AB/+	2	13.33		0	0.00			
O/+	4	26.67		1	14.29			
Antecedent pregnancy								1.000
Molar	3	20.00		2	28.57			
Term	5	33.33		3	42.86			
Abortion	5	33.33		2	28.57			
Ectopic	2	13.33		0	0.00			
Interval (months)			10 (30)			7 (70)	9.5 (34)	0.724
≤ 6	5	33.33		3	42.86			
7–12	3	20.00		2	28.57			
> 12	7	46.67		2	28.57			
LogHCG level (IU/L)			4.347 ± 1.202			4.602 ± 0.532	4.459 ± 0.969	0.651
≤ 5	11	73.33		5	71.43			
> 5	4	26.67		2	28.57			
Maximum diameter (cm)								0.384
< 5	9	60.00		3	42.86			
≥ 5	6	40.00		4	57.14			
Number of tumor lesions								0.015
≤ 8	11	73.33		1	14.29			
> 8	4	26.67		6	85.71			
Metastatic sites								1.000
Lung or reproductive organs and other organs	11	73.33		6	85.71			
Only special sites	4	26.67		1	14.29			
FIGO score			10.00 ± 4.472			16.75 ± 3.403	12.50 ± 4.964	0.197
≤ 12	5	33.33		1	14.29			
≥ 13	2	13.33		3	42.86			
Chemoresponse								<0.0001
Sensitive	14	93.33		0	0.00			
Resistant	0	0.00		6	85.71			
Dependent	1	6.67		1	14.29			
Primary chemotherapy regimen is EMA-CO or EP-EMA								0.014
Yes	13	86.67		2	28.57			
No	2	13.33		5	71.43			
Surgery								0.616
Yes	10	66.67		6	85.71			
No	5	33.33		1	14.29			
Pathological pattern								1.000
Choriocarcinoma	5	33.33		3	42.86			
ETT	2	13.33		1	14.29			
PSTT	1	6.67		1	14.29			
Invasive mole	2	13.33		1	14.29			
Coexisting	1	6.67		0	0.00			

Distant metastatic sites of all 26 stage IV GTN patients included the lung (n = 18, 69.2%), brain (n = 14, 53.8%), liver (n = 8, 30.8%), gastrointestinal tract (n = 4,15.4%), kidney (n = 3, 11.5%), spleen (n = 3, 11.5%), pancreas (n = 2, 7.7%) and adrenal gland (n = 1, 3.8%). Other rare sites included gallbladder, pleura, skin, etc. Besides the patients with lung metastases or with reproductive lesions, only 5 patients (19.2%) presented with isolated other distant organs metastases. According to statistics, 15 patients (57.7%) were sensitive to chemotherapy, 6 patients (23.1%) were resistant and 5 patients (19.2%) were dependent. 18 patients (69.2%) were primarily treated with first-line EMA-CO or EP-EMA chemotherapy regimen. Furthermore, 19 (73.1%) of the total 26 stage IV GTN patients underwent surgery before or after primary chemotherapy.

Table 3 showed clinical characteristics of 5 patients with special type in stage IV GTN. 4/5 (80%) patients were < 40 years old; 4/5 (80%) patients occurred after nonmolar pregnancy; 4/5 (80%) patients’ pathological pattern was choriocarcinoma; 4/5 (80%) patients had no evidence of GTN at least 2 years after all treatment.

**Table 3:** Demographic and clinical details of the 5 GTN patients only with special sites lesions

NO./Age	Antecedent pregnancy	Interval (months)	HCG level before chemotherapy (IU/L)	Metastatic sites	Number of lesions	FIGO score	Pathological pattern	Treatment	Status
1/17	Abortion	7–12	227.4	Brain	1	7	Choriocarci-noma	Craniotomy EMA-CO 5courses (HCG was normal after 2nd course)	NED
2/27	Term	< 4	> 100,0000	Pleura, liver, small intestine	> 8	16	Choriocarci-noma	EMA-CO 5curses EP-EMA 3courses. HCG level was not normal.(Giving-up chemotherapy, follow up to normal)	NED
3/34	Molar	< 4	19,453	Greater omentum	2	3	Invasive mole	MTX mono-chemotherapy 5courses (HCG was normal after 2nd course)	NED
4/46	Term	> 12	62,662	Liver, intestine	> 8	17	Choriocarci-noma	EMA-CO 5 courses (resistant), and changed to FAEV 1 course (HCG continued to rise) “Taxol + Oxaliplatin + Gemcitabine” 1 course (HCG continued to rise) TEP 7 courses Radiofrequency ablation (RFA) of hepatic lesions Hysterectomy + bilateral salpingectomy + partial colectomy BEP 3 courses MBE 1 course; EP-EMA 2 courses; PD-1 immunotherapy 2 courses; Resection of liver cancer; PD-1 immunotherapy 7 courses; TP 1 course	Died of multiple drugs resistant and multiple metastases
5/26	Term	>12	6217	Brain	2	10	Choriocarci-noma	Craniotomy EMA-CO 9courses (HCG was normal after 3rd course)	NED

*MTX* methotrexate, *FAEV* florouracil/floxuridine, etoposide, dactinomycin, vincristine, *TEP* taxol, etoposide, platinum, *BEP* bleomycin, etoposide, cisplatin, *MBE* methotrexate, bleomycin, etoposide, *PD*-*1* programmed cell death protein 1, *TP* taxol, platinum

### Treatment, outcomes and prognostic factors

Table 4 summarizes treatment regimens and outcomes in 26 stage IV GTN patients. Intravenous and intrathecal single agent or multidrug chemotherapy was individualized after multidisciplinary team meeting. Concerning primary initial chemotherapy, the EMA-CO protocol was used for 15 patients (57.7%), 10 of which with the NED status (66.7%), EP-EMA protocol was used for 3 patients (11.5%), all of them were complete response. MTX, FAEV, TP and ACM protocols once each. The FA protocol was used for 4 patients, 3 of them were dead and one was lost in the end. Table 2 revealed that primary chemotherapy regimen was EMA-CO or EP-EMA protocol could significantly improve prognosis (P = 0.014). 18 patients underwent surgical treatment before or after chemotherapy. The management including hysterectomy, neurosurgery, lesionectomy to liver, gastrointestinal tract, lungs, lymph nodes and so on. However, whether the patients underwent surgery or not, there was no significant effect on the results (Table 2, P = 0.616). Half of involved patients had further salvage chemotherapies, cranial local radiotherapy and immunotherapy.

**Table 4:** The overall treatment for 26 patients with stage IV GTN

Initial chemotherapy	Salvage treatment	Surgery	Status
TP	Cranial local radiotherapy, Apatinib	Hysterectomy, craniotomy	Dead
EP-EMA	Cranial local radiotherapy	Hysterectomy, lesionectomy	NED
FAEV	EMA-CO, EP-EMA, TP	Lesionectomy	NED
EMA-CO	–	–	NED
EMA-CO	–	Craniotomy	NED
EMA-CO	–	–	NED
EMA-CO	EP-EMA	–	NED
EMA-CO (intrathecal)	–	–	Under treatment
FA	EP-EMA, FAEV	Hysterectomy	Lost
EMA-CO	FCA, EP-EMA	Hysterectomy	Lost
MTX	–	Lesionectomy	NED
FA	EMA-CO	Hysterectomy, lesionectomy	Dead
EMA-CO	Cranial local radiotherapy, FEP, EP-EMA	–	Dead
EP-EMA	–	Lesionectomy	NED
FA	EMA-CO, EP-EMA, TP	–	Dead
EMA-CO	–	Hysterectomy	NED
EMA-CO	FAEV, TP, TEP, BEP, MBE, EP-EMA, immunotherapy	Hysterectomy, lesionectomy	Dead
EMA-CO	–	Craniotomy	NED
EMA-CO	–	Lesionectomy	NED
ACM	EP-EMA, TP	Hysterectomy	Dead
EMA-CO	EP-EMA	–	NED
FA	Cranial local radiotherapy, immunotherapy	Hysterectomy	Dead
EMA-CO	–	Craniotomy	NED
EP-EMA	–	Hysterectomy	NED
EMA-CO	EP-EMA, TP	Hysterectomy	Under treatment
EMA-CO	–	–	NED

*FA* 5-fluorouracil, actinomycin-D, *FCA* 5-fluorouracil, cyclophosphamide, actinomycin-D, *FEP* floxuridine, etoposide, cisplatin, *ACM* actinomycin-D, cyclophosphamide, methotrexate

According to Table 2, univariate correlation analyses focused on several prognostic factors. There were no significant differences in age, blood type, antecedent pregnancy type, the interval from last known pregnancy, pretreatment serum HCG level, maximum diameter of tumors, metastatic sites, FIGO score, underwent surgery or not and pathological pattern by the outcomes (P > 0.05). In other words, there was a non-significant difference in outcomes between patients with FIGO stage IV GTN and its special type. Number of tumor lesions (P = 0.015), chemotherapeutic response (P < 0.0001), primary chemotherapy regimen was EMA-CO or EP-EMA protocol or not (P = 0.014) were all significant in terms of effects.

Furthermore, univariate and multivariate logistic analyses of total involved variables were used to assess the prognostic significance. Interestingly, as Table 5 showed, the univariate analysis found a significant association between age (< 40 vs ≥ 40 years) and the outcome[odds ratio (OR) 6.533, 95% CI 1.200–35.573, P = 0.03]. Number of tumor lesions (≤ 8 vs > 8, P = 0.003), primary chemotherapy regimen was EMA-CO or EP-EMA protocol (yes vs no, P = 0.004) affected the prognosis significantly. Due to the small sample size of this study, chemoresponse was unable to enter regression analyses, other variables all had P value over 0.05. According to the multivariate logistic regression analysis, as Table 6 revealed, only number of tumor lesions > 8 (OR 23.714, 95% CI 1.394–403.296, P = 0.029) and primary chemotherapy regimen was not EMA-CO or EP-EMA protocol (OR 0.049, 95% CI 0.003–0.744, P = 0.03) were independent poor prognostic factors.

**Table 5:** Univariate logistic analysis of the association with outcomes

Variable	OR	95% CI	P value
Age (< 40 VS ≥ 40 years)	6.533	1.200–35.573	0.03
Number of tumor lesions (≤ 8 VS > 8)	35.750	3.465–368.829	0.003
Primary chemotherapy regimen is EMA-CO or EP-EMA (Yes VS No)	0.062	0.009–0.406	0.004

All variables were included, here’s presented variables P < 0.05

**Table 6:** Multivariate logistic analysis of the association with outcomes

Variable	OR	95% CI	P value
Age (< 40 VS ≥ 40 years)	4.360	0.245–77.554	0.316
Number of tumor lesions (≤ 8 VS > 8)	23.714	1.394–403.296	0.029
Primary chemotherapy regimen is EMA-CO or EP-EMA (Yes VS No)	0.049	0.003–0.744	0.03

### Assessment of chemoresponse

Table 7 summarizes clinical characteristics predicting chemoresponse. In this study, age, blood type, antecedent pregnancy type, the interval from last known pregnancy, pretreatment serum HCG level, maximum diameter of tumors, metastatic sites, FIGO score, underwent surgery or not and pathological pattern all did not significantly affect the response to chemotherapy. The patients having tumor lesions ≤ 8 were much more sensitive to chemotherapy compared to the group having tumor lesions > 8 (78.6% vs 33.3%, P = 0.007). Besides, the patients using EMA-CO or EP-EMA regimen as the initial chemotherapy protocol had greater sensitive rate of treatment than not (77.8% vs 12.5%, P = 0.002).

**Table 7:** Univariate correlation analyses of chemoresponse in gestational trophoblastic neoplasia patients with FIGO stage IV and its special type

Variables/categories	Chemoreponse (no. of patients)			P value
	Sensitive	Resistant	Dependent	
Age (years)				0.146
< 40	12	2	3	
≥ 40	3	4	2	
Blood type				0.340
A/+	4	3	3	
B/+	5	2	1	
AB/+	4	0	0	
O/+	0	1	1	
Antecedent pregnancy				1.000
Molar	3	2	1	
Term	5	2	2	
Abortion	5	2	2	
Ectopic	2	0	0	
Interval (months)				0.940
≤ 6	5	3	1	
7–12	3	1	1	
> 12	7	2	3	
LogHCG level (IU/L)				0.707
≤ 5	11	5	3	
> 5	4	1	2	
Maximum diameter (cm)				0.563
< 5	9	2	3	
≥ 5	6	4	2	
Number of tumor lesions				0.007
≤ 8	11	0	3	
> 8	4	6	2	
Metastatic sites				0.792
Lung or reproductive organs and other organs	11	5	5	
Only special sites	4	1	0	
FIGO score				0.242
≤ 12	5	1	0	
≥ 13	2	3	1	
Primary chemotherapy regimen is EMA-CO or EP-EMA				0.002
Yes	14	1	3	
No	1	5	2	
Surgery				0.167
Yes	9	6	4	
No	6	0	1	
Pathological pattern				0.994
Choriocarcinoma	4	3	2	
ETT	2	1	1	
PSTT	1	1	0	
Invasive mole	2	1	0	
Coexisting	1	0	1	

### IHC features

9 patients’ immunohistochemical staining results had been collected (Table 8). HCG was present in almost 100% choriocarcinoma, ETT and PSTT. P63 was stronger expressed in ETT. Two choriocarcinoma complicated with ETT were negative in the expression of SALL4. Patients with P53 positivity had worse outcomes than their negative expression.

**Table 8:** The immunohistochemical (IHC) features of 9 patients

NO./age	GTN pathological type	IHC report	Status
1/26	ETT	HCG−, HPL+/+−, P63++, P53−	NED
2/28	ETT	HCG+, HPL+	Lost
3/36	Choriocarcinoma, ETT	HCG scattered+, HPL scattered+, P63++, SALL4−	NED
4/40	Choriocarcinoma	HCG+, HPL−	Dead
5/45	ETT	HCG extensive+, HPL+, P53−	NED
6/26	Choriocarcinoma	HCG++, HPL+++, P63−	NED
7/42	PSTT	HCG+, HPL+, P63+	NED
8/48	Choriocarcinoma, ETT	HCG+, HPL partial+, P63 partial+, SALL4−	Under treatment after recurrence(sensitive to chemotherapy)
9/46	Choriocarcinoma	HCG+, HPL partial+, P53 90%++, p63 little+	Dead

*HPL* human placental lactogen

### Survival analyses

**Fig. 1:**
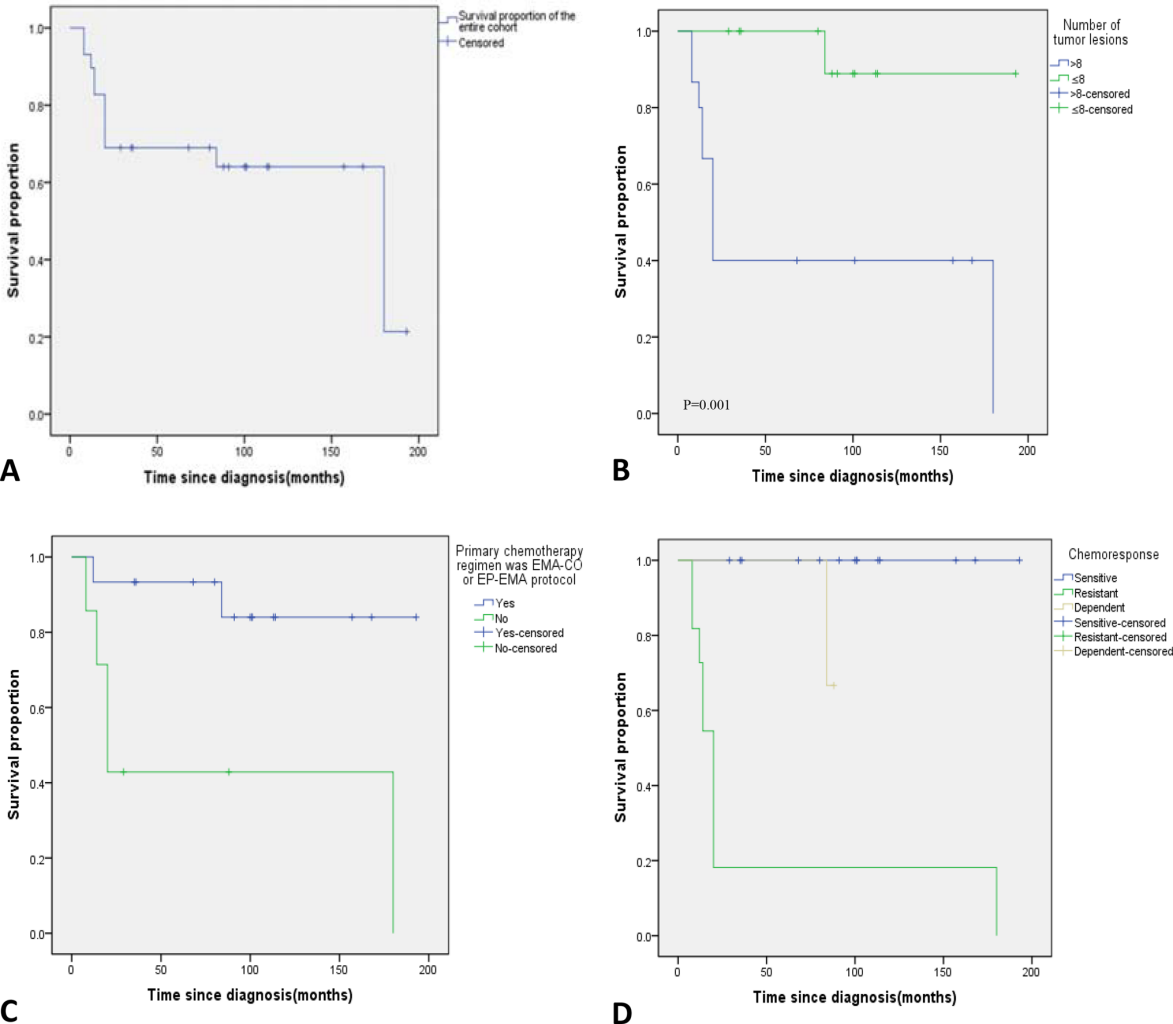
Kaplan-Meier curve for **a** survival proportion of the entire cohort, **b** survival of patients with tumor lesions ≤ 8 versus > 8, **c** survival of patients whether or not treated with EMA-CO or EP-EMA as the primary chemotherapy regimen, **d** survival of patients with sensitive versus resistant versus dependent chemoresponse

As shown in Fig. 1a, The 5-year OS rate of the total 26 FIGO stage IV GTN patients was 69.0%. There was a significant association between number of tumor lesions (P = 0.001), primary chemotherapy regimen was EMA-CO or EP-EMA protocol (P = 0.005), chemoresponse (P < 0.0001) and survival. Patients with tumor lesions of > 8 had a significantly poorer OS than those with tumor lesions of ≤ 8 (Fig. 1b). Patients who used EMA-CO or EP-EMA as the initial chemotherapy regimen showed significantly higher OS than not (Fig. 1c).

Table 9 showed multivariate COX regression analyses to assess the predictive significance of OS. All variables had been tested by univariate and multivariate COX proportional hazards regression analyses. Number of tumor lesions, primary chemotherapy regimen was EMA-CO or EP-EMA protocol or not, chemoresponse were found as influence factors for OS in univariate analysis. Only number of tumor lesions > 8[HR 0.101, 95% CI 0.011–0.900, P = 0.040] was independent prognostic factors associated with poorer OS.

**Table 9:** Multivariate Cox proportional regression of the association with outcomes

Variable	HR	95% CI	P value
Age (< 40 VS ≥ 40 years)	1.481	0.379–5.785	0.572
Number of tumor lesions (≤ 8 VS > 8)	0.101	0.011–0.900	0.040
Primary chemotherapy regimen is EMA-CO or EP-EMA (Yes VS No)	3.674	0.702–19.239	0.123

### Comment

As the results above showed, patients with FIGO stage IV GTN are rare, its special types are extremely rare. For the first time, we found that there is no significant difference of survival rate between stage IV GTN and its special type. The CR rate and 5-year OS rate were relatively high in our study. Reported by other institutions at home and abroad, the incidence of stage IV GTN was 8.1–12.0% [6, 15, 16]. Research of recent 10 years showed that the complete remission rate was 50.0–80.0%, and the overall survival rate was 62.0–93.3% [4, 6, 17]. That might depend on several factors: the distribution of prevalence rate in different areas and races, medical institutions’ ability and experience of treatment, in addition, the using of therapeutic regimens varied from person to person.

Jun Li et al. reported that EMA-CO, EP-EMA and FAEV were the three most commonly used initial chemotherapy regimens, respectively, the CR rate was 55.2%, 60.0% and 63.1% [18]. In our study, most patients with choriocarcinoma treated with EMA-CO as primary chemotherapeutic protocol, only one treated with FAEV, while patients diagnosed with ETT or PSTT were mainly initially treated with EP-EMA regimen. This study indicated that patients primarily treated with EMA-CO or EP-EMA protocol had much better outcome.

Since the rarity of stage IV GTN, the associated prognostic factors remain controversial. In the present study, univariate analyses showed that age < 40, number of tumor lesions ≤ 8, primary chemotherapy regimen was EMA-CO or EP-EMA protocol and patients sensitive to chemotherapy were predictors of better prognosis. Multivariate analyses revealed that only number of tumor lesions > 8 was independent risk factor for predicting a poor survival. For patients with FIGO stage IV GTN, type of antecedent pregnancy, pretreatment serum β-HCG levels, FIGO score, interval from last known pregnancy, pathological pattern, maximum diameter of tumors, blood type and had surgery or not were not significantly associated with survival. Interestingly, in our study, there were different results of association between age and outcomes with different statistical analyses methods. We confirmed that age < 40 is an influence factor with negative prediction value. However, we have to point out that there were some limitations in this study. First of all, due to the insufficient number of cases, chemoresponse was unable to enter logistic or COX regression analyses, we did not analyse the response to different chemotherapy regimens, moreover, this might lead to no differences of outcomes between each kind of pathological patterns. Secondly, we did not explore in detail about if patients’ last known pregnancy was the causative pregnancy, which might reveal the totally opposite correlation analyses result. Therefore, we got something similar and something in contrast compared with the studies of Jiang Fang et al. [3], Eysbouts et al. [12] and Yang et al. [4]. Last but not least, about the diagnosis of special type stage IV GTN, there was the possibility of misdiagnosis due to medical examination errors, which might lead to different results. But we minimized errors as far as possible. Our key strength lied in that the paper summarized the characteristics of patients with special type stage IV GTN for the first time.

According to the characteristics of 5 patients with special stage IV GTN. We tentatively put forward that the group with age less than 40 years old, patients with nonmolar antecedent pregnancy and patients diagnosed with choriocarcinoma have greater incidence of special type of stage IV GTN. Furthermore, we believe that the patients have high response rate of chemotherapy and have favorable prognosis as a whole. In our study, there was no significant difference in chemotherapeutic response between FIGO stage IV GTN and its special types. As for chemoresponse, we investigated the correlated influence factors. Our data suggested that patients with tumor lesions number ≤ 8 were much more sensitive to chemotherapy. Using EMA-CO or EP-EMA regimens as the initial chemotherapy protocol showed much better effectiveness.

Given the limited usefulness of surgery, available histopathological specimens were really few. Because of the different therapeutic protocols, it is necessary to distinguish choriocarcinoma from ITTs, besides, special types of stage IV GTN must be differentiated from other malignancies (primary or after spreading). Many researchers have been studying the relatively specific GTN tumor markers based on existing pathological data. Not just for identification after surgery, if such kind of tumor markers can be found in blood, patients can be treated with more accurate chemotherapy regimens early in the illness, even adopt precise targeted immunotherapy. Our data suggested that none of HCG and HPL specifically expressed, P63 was stronger expressed in ETT. Similarly, Morgane Stichelbout et al. [19] also raised that HCG, HPL and P63 were detected in choriocarcinoma, PSTT and ETT, but P63 was diffusely expressed in ETT. In addition, they demonstrated that SALL4 is specifically expressed in choriocarcinoma, not in PSTT and ETT. Each type of gestational trophoblastic tumors can coexist. As our data showed, two choriocarcinoma complicated with ETT were negative in the expression of SALL4. Pengming Sun et al. [20] reported that the expression levels of mutant P53 were increased with the development of GTN. The information in our study likewise indicated that patients with P53 positivity had worse outcomes than its negative expression. Nonetheless, the current study was small, these conclusions still require further validation.

## Conclusions

In summary, patients with FIGO stage IV GTN and its special type all have favorable prognosis if they have early certain diagnosis and appropriate therapy schedule. The findings of the research suggested that patients with age less than 40 years, or with number of tumor lesions ≤ 8, or treated with one of EMA-CO, EP-EMA and FAEV regimens as the initial chemotherapy protocol have relatively better survival outcomes. Some patients with special type of stage IV GTN visit the relevant departments due to the symptoms corresponding to different metastatic sites in various organs. It is crucial to have the correct diagnosis. For childbearing women, who are especially suspected of having hemorrhagic malignant metastases, serum HCG level must be primarily tested. In addition, owing to different treatment strategies, it is really important for the differential diagnosis of this disease and the primary or metastatic carcinoma in other organs. We confirmed that surgery plays a limited role in the cure rate of stage IV GTN. However, for patients with acute signs or have tumor lesions > 8, surgery is the first selection to save lives and decrease the tumor loads, which can significantly improve chemosensitivity and prognosis in the meanwhile. Owing to the rarity of the cases with only special sites metastases from GTN, it is necessary to conduct large retrospective studies aimed at exploring the diagnosis, treatment and outcomes of this subgroup patients. It is necessary to further explore the specific tumor markers of GTN, which must be found in blood, so that patients can be treated with more accurate chemotherapy regimens early in the illness, even adopt precise targeted immunotherapy before surgery.

## Electronic supplementary material

Below is the link to the electronic supplementary material.

Supplementary material 1 (DOCX 21 kb)

## Data Availability

All data generated or analysed during this study are included in this published article.
