# Supplementary material for: Management and prognostic analysis of patients with gestational trophoblastic neoplasia (GTN) in FIGO stage IV and its special type
Source: Clin Exp Metastasis. 2020 Nov 20;38(1):47–59. doi: 10.1007/s10585-020-10064-w (PMC7882548; doi:10.1007/s10585-020-10064-w)
Supplement: Supplementary file 1 — Supplementary material 1 (DOCX 21 kb) [file 10585_2020_10064_MOESM1_ESM.docx]

| Table10. A summary of the NED patients’ characteristics | | | | | | | |
| --- | --- | --- | --- | --- | --- | --- | --- |
| No./age | Antecedent  Pregnancy | Blood  Type  (ABO/Rh) | HCG level before treatment  (IU/L) | Interval from termination | Lesion  sites | FIGO score^a^ | Treatment  Protocols(in chronological order) |
| 1/26 | Abortion | B/+ | 50518 | 36 months | Lungs,brain, Lymph nodes | - | 1.EP-EMA 8 courses  (2 cycles of consolidation therapy)  2.Brain radiotherapy  3.Hysterectomy+bilateral salpingectomy+lymph nodes biopsy |
| 2/33 | Term | A/+ | 95897 | 12+ months | Lungs,kidney, pleura, pelvic | - | 1.”5-FU+KSM+VCR” 6 courses  2.EMA-CO 4 courses  (2 cycles of consolidation therapy)  3.EP-EMA 6 courses  (3 cycles of consolidation therapy)  4.TP 5 courses  (3 cycles of consolidation therapy)  Multiple recurrence  5.Dissected the lung lesions |
| 3/27 | Ectopic | - | 17940 | 2 months | Ovary,pelvic, mesenterium, stomach lesser omenrum | - | 1.EMA-CO 4 courses  (2 cycles of consolidation therapy) |
| 4/17 | Abortion | A/+ | 227.4(after operation ) | 10 months | Brain | 7 | 1.Craniotomy  2.EMA-CO 5 courses  (3 cycles of consolidation therapy) |
| 5/41 | Molar | A/+ | 23282 | 1 month | Uterine,vaginal, lung, brain | 9 | 1.EMA-CO 8 courses  (4 cycles of consolidation therapy) |
| 6/27 | Term | AB/+ | >100 0000 | 2 months | Liver,pleura, small bowel | 16 | 1.EMA-CO 5 courses  2.EP-EMA 3 courses  (HCG was not yet normal after chemotherapy) |
| 7/34 | Molar | B/+ | 19453 | 15 days | Pelvic,greater omentum | 3 | 1.Lesionectomy  2.MTX 5 courses  (3 cycles of consolidation therapy) |
| 8/36 | Abortion | O/+ | 128.8(after operation ) | 32 months | Ovary,greater omentum,Abdominal wall | - | 1.Lesionectomy  2.EP-EMA 4 courses  (3 cycles of consolidation therapy) |
| 9/45 | Abortion | O/+ | 20787 | 8 months | Uterine,lung, pancreas | - | 1.Hysterectomy+bilateral salpingectomy  2.EMA-CO 9 courses  (3 cycles of consolidation therapy) |
| 10/21 | Molar | O/+ | 665.6(after operation ) | 36 months | Lung,brain | 10 | 1.Crainotomy  2.EMA-CO 6 courses  (3 cycles of consolidation therapy) |
| 11/26 | Ectopic | AB/+ | 910474 | During pregnancy | Uterine,lung,liver,intestine,greater omentum,peritoneum,muscle,etc | 15 | 1.Cesarean section+lesionectomy  2.EMA-CO 3 courses  (HCG was not yet normal after chemotherapy) |
| 12/26 | Abortion | B/+ | 587820 | 12+ months | Uterine,lung, renal,spleen | - | 1.EMA-CO 5 courses  2.EP-EMA 4 courses  (3 cycles of consolidation therapy) |
| 13/26 | Term | A/+ | 6217(after operation ) | 9 months | Brain | 10 | 1.Crainotomy  2.EMA-CO 9 courses  (6 cycles of consolidation therapy) |
| 14/42 | Term | O/+ | 19087 | 60 months | Uterine,lung,pancreas | - | 1.Uterine curettage  2.EP-EMA 2 courses  3.Hysterectomy+bilateral salpingectomy  4.EP-EMA 5 courses  (2 cycles of consolidation therapy) |
| 15/31 | Term | A/+ | 156094 | 20 months | Uterine,lung, brain, muscle | - | 1.EMA-CO 6 courses  (1 cycles of consolidation therapy) |
| 5-FU: 5-fluorouracil; KSM: kasugamycin; VCR: vincristine  ^a^: The FIGO prognostic scoring system was inapplicable to patients with ITT. | | | | | | | |

| Table11. A summary of the dead patients’ characteristics | | | | | | | |
| --- | --- | --- | --- | --- | --- | --- | --- |
| No./age | Antecedent  Pregnancy | Blood  Type  (ABO/Rh) | HCG level before treatment  (IU/L) | Interval from termination | Lesion  sites | FIGO score | Treatment  protocols |
| 1/46 | Molar | A/+ | 38000 | <4 months | Uterine,lung, brain,hepatic, stomach, spleen, abdominal wall, muscle,etc | - | 1.Hysterectomy+bilateral salping-oophenrectomy+Pelvic Lymphyadenectomy  2.TP 2 courses  3.Brain radiotherapy  4.Apatinib (cerebral hemorrhage occurred)  5.Craniotomy  Died of cerebral hemorrhage |
| 2/29 | Term | B/+ | 23948 | 8 months | Uterine,lung, brain,hepatic, lymph nodes | - | 1.”5-Fu+Act-D” 1 course  2.EMA-CO 3 courses  3.Hysterectomy+bilateral salpingectomy+Pelvic Lymphyadenectomy  4.EMA-CO 2 courses  5.Resection of metastatic liver cancer  6.EP-EMA 1 course  Died of cerebral hemorrhage |
| 3/29 | Term | B/+ | >20 0000 | 7+ months | Lung,brain | - | 1.EMA-CO 9 courses  2.Brain radiotherapy  3.FEP 3 courses  4.EP-EMA 4 courses  Died of repeated recurrence and systemic failure |
| 4/40 | Abortion | B/+ | 10714 | 6 years | Lung,brain,skin | 20 | 1.MTX 2 courses  2.”5-FUDR+KSM” 2 courses  3.EMA-CO 2 courses  4.EP-EMA 3 courses  5.TP 2 courses  6.”Gemcitabine+cisplatin”3courses  Died of multiple drugs resistant and systemic failure |
| 5/54 | Molar | A/+ | 155378 | 1+ month | Uterine,lung, brain,liver | 18 | 1.Hysterectomy+right salping-oophenrectomy  2.ACM 4 courses  3.EP-EMA 8 courses  4.TP 5 courses  5.EMA 8 courses  6.TP 3 courses  Died of repeated recurrence and systemic failure |
| 6/37 | Abortion | O/+ | 8548 | 1 month | Uterine,lung, brain | 12 | 1.”KSM+5-Fu” 1 course  2.Hysterectomy+left salping-oophenrectomy  3.Brain radiotherapy  4.Taxol monotherapy for 1 course  5.”KSM+MTX+AT 1258” 4 courses  6.”AT 1258+VP 16-213” 2 courses  7.PVB 1 course  8.”Thymopentin,IL-2,IFN” immunotherapy  9.PVB 3 courses  10.EMA-CO 3 courses  11.”Oxaliplatin+5-Fu+CF/FA” 1 course  12.EMA-CO 1 course  Died of multiple drugs resistant, repeated recurrence and systemic failure |
| 7/46 | Term | A/+ | 62662 | 14years | Liver, intestine | 17 | 1. EMA-CO 5 courses(resistant), and changed to FAEV 1 course(HCG continued to rise) 2. “Taxol+Oxaliplatin+Gemcitabine” 1 course(HCG continued to rise) 3. TEP 7 courses 4. Radiofrequency ablation(RFA) of hepatic lesions 5. Hysterectomy+bilateral salpingectomy+partial colectomy 6. BEP 3 courses 7. MBE 1 course; EP-EMA 2 courses; PD-1 immunotherapy 2 courses; Resection of liver cancer; PD-1 immunotherapy 7 courses; TP 1 course |
| Act-D: actinomycin-d; 5-FUDR: 5-fluoro-2'-deoxyuridine; VP 16-213: etoposide; AT 1258: nitrocaphane; PVB: platinum, [vincristine](E:/Dict/8.6.2.0/resultui/html/index.html#/javascript:;), bleomycin; IL-2: interleukin-2; IFN: interferon; CF/FA: calcium folinate/[fludarabine](E:/Dict/8.9.3.0/resultui/html/index.html#/javascript:;); PD-1: programmed cell death protein 1 | | | | | | | |
